# Beyond Hemoglobin A1c—Outcomes That Matter to Individuals With Type 1 Diabetes in Adopting Digital Health Interventions for Self-Management Support: Qualitative Study

**DOI:** 10.2196/60190

**Published:** 2024-11-07

**Authors:** Benjamin Markowitz, Stephanie de Sequeira, Adhiyat Najam, Cheryl Pritlove, Dana Greenberg, Marley Greenberg, Chee-Mei Chan, Gurpreet Lakhanpal, Samyukta Jagadeesh, Geetha Mukerji, Rayzel Shulman, Holly O Witteman, Catherine H Yu, Gillian L Booth, Janet A Parsons, The T1ME Patient Advisory Committee

**Affiliations:** 1 Unity Health Toronto Toronto, ON Canada; 2 University of Limerick School of Medicine Limerick Ireland; 3 University of Toronto Toronto, ON Canada; 4 Diabetes Action Canada Toronto, ON Canada; 5 University Health Network Toronto, ON Canada; 6 Trillium Health Partners Mississauga, ON Canada; 7 Women's College Hospital Toronto, ON Canada; 8 The Hospital for Sick Children Toronto, ON Canada; 9 Université Laval Quebec City, QC Canada; 10 VITAM Research Centre for Sustainable Health Quebec City, QC Canada; 11 CHU de Québec-Université Laval Quebec City, QC Canada; 12 Ottawa Hospital Research Institute Ottawa, ON Canada

**Keywords:** T1D self-management, patient reported outcomes, patient reported outcome measures, virtual care, mobile phone, type 1 diabetes

## Abstract

**Background:**

Type 1 diabetes is a demanding chronic condition that requires diligent blood glucose monitoring and timely insulin administration by patients who must integrate self-management into their daily lives.

**Objective:**

This study aimed to better understand what outcome measures are important to individuals living with type 1 diabetes (T1D) in Ontario, Canada, to help inform the development of type 1 diabetes virtual self-management Education and support (T1ME) trial.

**Methods:**

A qualitative approach was used, in which we conducted 6 focus groups with a total of 24 adult participants living with T1D (from age 18 to >65 years) in Ontario. Each focus group was semistructured in nature; participants were encouraged to talk openly about their experiences with T1D self-management and provide their perspectives on more focused topics such as technology and relationships with health care providers.

**Results:**

An interpretive analysis helped us devise a framework for our results that centered around 6 main discussion themes: (1) adapting self-management to meet evolving needs, (2) looking “beyond A1c” toward more personalized indicators of glycemic management, (3) the benefits and challenges of adopting new T1D technology, (4) establishing trusting relationships with diabetes care providers, (5) perceived benefits of peer support, and (6) pre– and post–COVID-19 perspectives on virtual care.

**Conclusions:**

Our goal is for these findings to help facilitate the development of patient-oriented outcome measures that are in line with the unique needs and preferences of T1D patients in this new, more virtual landscape of clinical care, education, and self-management support.

## Introduction

Type 1 diabetes (T1D) is one of the most complex chronic conditions to manage on a daily basis, requiring constant vigilance through self-monitoring of glucose levels and moment-to-moment decision-making regarding insulin dosing. To monitor an individual’s glycemic control and risk for long-term complications, hemoglobin A_1c_ (HbA_1c_) is the gold standard biometric used in diabetes practice [1-3]. Given the demanding nature of self-management, receiving a poor HbA_1c_ result can be discouraging for those living with T1D [4,5], which can lead to feelings of guilt [6], burnout, loss of motivation, and diabetes distress [7,8]. In addition to HbA_1c_, there has been a greater shift toward using time in range in diabetes care [9], which represents the percentage of one’s time spent with normal or near-normal glucose levels as encapsulated by data from continuous glucose monitoring (CGM) [10]. This continuous metric of glycemic control is helpful for providers to facilitate meaningful dialogue with individuals living with T1D and helps clarify what may be affecting their glycemic management outside of the clinic [11].

The work associated with T1D self-management has been compared with a 7-day-a-week, 24-hour-a-day job that involves diligent blood glucose monitoring and frequent decision-making to match insulin administration with dietary intake [12,13]. In addition, individuals living with T1D have to consider the impact that other aspects of daily life have on their glycemic management, including work schedules, exercise regimens, sleep, and stress [14]. Due to the “24/7” nature of self-managing T1D, there is immense potential for improvements in glycemic management associated with digital health interventions that promote frequent communication and facilitate peer mentorship and support (eg, text messaging, emails, and videoconferencing) [15-18]. Indeed, online care has become more commonplace in T1D care and is routinely available to patients in Canada [19], but we are still learning how to deliver virtual care in a way that best meets patients’ unique needs.

In our qualitative study, we set out to address the research question, that is, what outcomes matter to patients with T1D and what would make them want to adopt new virtual care technology? To address this question, we facilitated open-ended dialogue with participants in the context of both in-person and virtual focus groups. These focus group discussions were moderated using a focus group guide (Multimedia Appendix 1) that helped participants converse openly about their lived experiences of T1D self-management, as well as their unique education and support needs, including their perspectives on using virtual care. Our aim is that our findings and analysis can help inform the design of patient-reported experience measures (PREMs) and patient-reported outcome measures (PROMs) [20] in the Type 1 diabetes virtual self-Management Education and support Trial (T1ME) trial in Ontario [21], and more broadly, help diabetes care providers better individualize their self-management support.

## Methods

### Context of the TIME Study

This qualitative study was undertaken to inform a randomized controlled trial, the T1ME Trial [21]. The T1ME Trial aims to test a high-frequency, low-touch (virtual) model of care for persons with T1D. Patient partnership is a core feature of the T1ME Trial, and the Patient Advisory Committee (PAC) has been contributing to its design and implementation. Early on, the PAC members commented that the planned primary outcome for the trial (HbA_1c_), struck them as problematic, given that many people living with T1D dislike being “defined” by their HbA_1c_ and HbA_1c_ cannot capture other, more relevant aspects of life with T1D. They asked us to explore what other outcomes might be relevant to people living with T1D, and how these inform their daily self-management practices, which was the impetus for this qualitative investigation. The goal of the study was to understand the types of outcomes that were perceived as meaningful by a diverse sample of adults living with T1D in Ontario, in the context of their usual care and self-management experiences. In order for the study to inform the trial’s design, we also needed to understand their education and support needs. In addition, we aimed to identify the aspects of a digital health care intervention that were important to individuals living with T1D.

### Qualitative Study Design

We sought to understand participants’ lived experiences managing T1D along with their perspectives on self-management support, education, and outcomes, with an emphasis on virtual care. To do this, we used an interpretive, qualitative methodology, which was exploratory in nature [22,23], and patient-centered in its design [24]. A thematic approach to qualitative analysis was adopted to generate themes based on focus group discussions [25]. Each focus group was semistructured in nature, in which a focus group guide contained open-ended and probing questions to help facilitate dialogue amongst participants. In this dialogical approach [26], we used our guiding questions to help frame discussions but we encouraged participants to convey their distinct lived experiences [26,27]. We intend for quotes to help define our themes in the analysis [25] and we also provide interpretations around similarities and diverging viewpoints conveyed through discussions amongst participants, such as *“information overload”* presented later on.

### Ethical Considerations

The study was reviewed and approved by the St. Michael’s Hospital, Unity Health Toronto Research Ethics Board (#19-201). All participants gave written informed consent.

### Sampling and Recruitment

Through a convenience sampling approach, we were able to include participants living with T1D from diverse sociodemographic positions, with a range of perspectives [28,29]. Eligible participants included Ontario residents 18 years of age or over living with T1D. Participants were recruited by BM from multiple sources, including a diabetes clinic in Toronto, internet-based study advertisements posted by a national diabetes organization (Diabetes Canada), and snowball sampling [29]. With the onset of the COVID-19 pandemic, participant recruitment became increasingly difficult; while we stopped at 6 focus groups, we feel that conceptual saturation was achieved, in that no further themes were being identified [28].

### Data Collection

We conducted focus groups with participants [30] either in-person (at St. Michael’s Hospital, Unity Health Toronto) or virtually using videoconferencing technology (Zoom Video Communications) between January 2020 and July 2020. The shift to virtual groups was necessitated by the onset of the COVID-19 pandemic, all focus groups conducted after March 1, 2020, were conducted virtually due to COVID-19 pandemic containment measures. Focus groups lasted from 60 to 120 minutes, were audio recorded and transcribed, and field notes were recorded. Participants also completed a demographic questionnaire.

We created a semistructured focus group guide without predetermined hypotheses, rather we wanted to ground our analysis in the discussions with participants [31] and identify patient-oriented outcomes that would reflect their unique perspectives and experiences [32]. The focus group guide was developed by an endocrinologist (GLB), researchers with previous experience in T1D (BM, JAP, and CP), and individuals with lived experience of T1D (ie, input from the Patient Advisory Committee; Multimedia Appendix 1). The focus group guide included open-ended questions regarding participants’ self-management experiences, along with questions regarding their use of various technologies, and any experiences with virtual care or incorporating technologies into their self-management. We also included a hypothetical scenario regarding a digital health (smartphone application) intervention. The objective of each focus group discussion was to encourage participants to describe their own experiences with self-management and elicit their perspectives on self-management education and support, delivered either virtually or in person. The moderators of the focus groups (BM and JAP) are individuals with expertise in qualitative research methodology, and who had previously conducted research on the topic of T1D.

### Data Analysis

Focus group transcripts were analyzed using an inductive, interpretivist approach [31], in which we developed a thematic framework that encapsulated our interpretations of the dataset [25]. We defined themes [31], looking for patterns within and across focus group transcripts [32,33]. Building themes from the perspective of participants [24,31], allowed us to devise a conceptual framework that portrays what life with T1D is like for them, along with commonalities and differences in their first-hand accounts.

During the course of analysis, we met periodically to discuss the evolving conceptual framework and link our findings to relevant literature [34]. These analytical meetings involved input from the whole analytical team (SD, BM, GLB, and JAP). In particular, we benefited from the guidance of our senior authors, which provided us with clinical insight from a practicing endocrinologist and health services researcher (GLB), along with methodological expertise in qualitative social science (JAP). We sought clarification of participants’ responses during the focus groups (question-answer technique) [35], in addition, we participated in discussions with our project’s patient partners, and we refined our interpretation of the results and our conceptual framework based on this feedback.

While our approach to qualitative analysis was inductive, interpretivist, and stayed close to participants’ accounts [22], our analysis was also informed by theory [26,27,36]*.* We drew on theory as we were analyzing the first-hand participant accounts to help make sense of the evolving qualitative dataset [36]. From this approach, we used theory for interpretive purposes to help build our thematic framework during analysis [22]. Our interpretations were rooted strongly in the notions of the “work” of chronic illness self-management [37], including the work entailed in managing T1D [13,14], and how technology might play a role in mitigating this work [38].

Given our practical focus on moving beyond HbA_1c_ toward (patient-centered) outcome measures that matter to people with T1D [7,9], social theory helped us think about how a multitude of biopsychosocial factors interplay in complex narratives about life with T1D, including how factors such as “A1c,” time in range, the T1D community and social relationships (eg, with family, friends, and health care providers) can influence one’s perspective on self-management. In particular, narrative theory [26,27] played a prominent role throughout the analysis, as we thought about how individual participant stories about the T1D experience, along with focus group dialogue, came together to illustrate broader themes related to the social context of living with T1D in Ontario. From this narrative stance [26,27], theory helped us think about what life with diabetes was like from the perspectives of participants and to better understand what challenges or aids their self-management outside of the clinic.

## Results

### Overview

From January to July 2020, we conducted 6 focus groups (5 virtual and 1 in-person) with a total of 24 participants (an average of 4 participants in each focus group). Table 1 outlines the demographic and clinical characteristics of participants. In terms of age, participants ranged from emerging and young adults (aged 18-30 years) to older adults (aged 65 years and older). Time living with T1D ranged from 1 to 56 years.

Our analysis identified 6 main themes from participant discussions around self-management, including (1) adapting self-management to meet evolving needs, (2) looking “beyond A1c” toward more personalized indicators of glycemic management, (3) the benefits and challenges of adopting new T1D technology, (4) establishing trusting relationships with diabetes care providers through holistic care, (5) perceived benefits of peer support, and (6) pre– and post–COVID-19 perspectives on virtual care. Each theme is discussed in detail below.

**Table 1:** Demographic and clinical characteristics of the participants.

Characteristic (N=24)			Values, n (%)
**Gender**			
	Men	9 (38)	
	Women	15 (62.5)	
**Age ranges** ^a^ **of participants (years)**			
	18 to 24; emerging adults	2 (8)	
	25 to 44; young adults	12 (50)	
	45 to 64; middle-aged adults	7 (29)	
	≥65; older adults	3 (13)	
**Duration of diabetes at focus group**			
	1 to 10 years	6 (25)	
	11 to 20 years	4 (17)	
	21 to 30 years	3 (12)	
	31 to 40 years	4 (17)	
	≥ 41 years	7 (29)	
**Insulin administration**			
	Continuous subcutaneous insulin infusion	17 (71)	
	Open artificial pancreas system (Closed Loop)	1 (4)	
	Multiple daily injections	7 (29)	
	Continuous glucose monitoring technology	21 (88)	
**Occupation**			
	Full-time student	2 (8)	
	Full-time work	14 (58)	
	Part-time work	2 (8)	
	Unemployed	2 (8)	
	Retired	3 (13)	
**Place of residence in Ontario**			
	Urban	23 (96)	
	Rural	1 (4)	

^a^Age ranges were classified based on literature looking at type 1 diabetes throughout distinct periods of adulthood [39].

### Adapting Self-Management Strategies to Meet Evolving Needs

This theme relates to the learning process, in which participants described an ongoing need to adapt diabetes self-management to their evolving needs. Participants described the complex and unrelenting work of self-management. Many participants faced ongoing struggles and challenges trying to keep their blood sugar levels within a target range,

I find it a bit of a struggle every day, to be honest… I do have low blood sugars in the early mornings, before I even wake up, pretty often.

P11

However, as people live longer with T1D, self-management began to feel more like a “habitual” or “natural” part of everyday life. Participants explained that they achieved a sense of confidence and “control” through an active learning process guided by regular support from their health care team:

I feel so much more in control of what I can control, in the last six months than let's say, the first six months, cause it was [a] big, big change, at my age [mid-forties at diagnosis]… at first, I used to see the nurse and the doctor…every month …I was just learning… reading, trying to understand this new disease and how to control it. So now, I feel, I don't need them as often.

P12

Soon after diagnosis, participants reported that they developed confidence for managing diabetes on their own, as they acquired lived experience. For example, one participant characterized people with T1D as “*ambitious people*” (P13) who have to acquire their own set of skills and expertise for self-management, a skill set they described as being unique from the diabetes education they received from health care providers.

Adaptation was a key part of this skill set, as participants constantly had to adjust to new challenges associated with changing life circumstances, such as adapting to parenthood, a new job, or beginning post-secondary school in a new city:

One of the challenges that I'm finding is that as we age, and go through different stages of life, we have to find ways to adapt to whatever’s changing. So, for instance… getting married, having children… not being able to focus solely on yourself, because you’re concerned about other people now… So it’s more about adapting to life as it changes.

P14

Participants portrayed living with T1D as a dynamic experience that occurs within a psychosocial context that fluctuates across the life course.

### Looking Beyond HbA_1c_ Toward Personally Meaningful Indicators of Glycemic Management

Looking beyond “A1c,” participants spoke about the benefits of using more nuanced outcomes, such as time-in-range, to assess their glycemic management. For example, participants discussed a desire to shift the conversation with providers away from HbA_1c,_ and recognized its many limitations,

A1c, it’s an average and it’s an average that doesn’t necessarily tell the truth. You could be consistently at seven or you can go … up and down like a yo-yo and then, it’ll still average it out to seven. It can still look like ‘Oh, you’re doing perfectly well.’ whereas you’re doing anything but well. So you can’t really count on the A1c alone.

P17

Some participants explained that they valued measures that were more relevant to their lived experience over more quantitative indicators such as HbA_1c_ or time-in-range. A key example related to the physical symptoms associated with high or low blood glucose levels (essentially how physically comfortable they felt throughout the course of a day). As one participant recounted,

I think a lot of it is just, I don’t want to be in discomfort. When I’m high, I’m uncomfortable; when I’m low, I’m uncomfortable.

P18

Participants also commented on how these symptoms interrupted aspects of their life such as job performance or being able to participate in hobbies.

### The Benefits and Challenges of Adopting New Type 1 Diabetes Virtual Self-Management Education and Support Technology

Participants reported using a range of technologies to manage their T1D, and to experience life without feeling as though diabetes was dominating their attention. One participant described how “looping” technology saved them from having to make a “hundred thousand decisions” each day [P7]. “Looping” refers to either commercial or open-source, community-developed closed-loop systems that use CGM readings and algorithms to automatically adjust insulin delivery from a pump [38]. Aside from looping, participants spoke about how they used different insulin pumps, glucose monitors, and smartphone apps. Many of the participants recounted the time when they switched from insulin injections to an insulin pump as a particularly memorable moment, one that made self-management feel easier, and gave them more freedom.

I’ve only been on the pump for about five years. And I’m thirty years into this, and I’m kicking myself, literally. I should have been on it ten years ago. Because the impact on my hemoglobin A1c and so forth… but more, it is more convenient … and it is just a real game changer, switching over to that pump.

P10

Participants also offered their perspectives on what outcomes they would like to see if or when adopting a (new) digital health intervention. In particular, they spoke about how willing they would be to take on a new self-management support and education application. A participant characterized it this way.

If I were about to take on another intervention and you’re calling it an app, I'd want to make sure that it’s integrated into everyday life, and not become another task. So, I'm not adding on to the maintenance, I'm either increasing the efficiency of the maintenance or replacing some of those tasks.

P10

This notion of easing the burden of decision-making was important to participants and would factor into their willingness to adopt a new smartphone application, for example. Indeed, when talking about their “ideal diabetes app,” many participants explained that they would love to use an application that adapted to their behavioral habits and lifestyle,

If I could have anything, in…an app or…in a dream world… it would be something …that would go “You usually go to the gym at six pm on Tuesday. It's three pm on Tuesday. Do you want to lower your basal?” Or … “You're often low during the night at three am, after you go to the gym… you know, stuff like that…to try and help me predict and be that little … angel on my shoulder.”

P18

In addition to describing what an “ideal” app would look like, participants reported on their experiences using a range of apps and technology, as well as accessing online information from a variety of different sources (social media and patient organizations). Although most participants described access to technology and rapid access to information as being very beneficial, some said they could feel overwhelmed at times, because of “information overload” associated with technologies. From this perspective, a participant felt that trying to be overly precise could result in a sense of guilt or failure with glycemic management.

I think knowing your exact blood sugar can play little games with your head, I guess. You know? “Oh crap, it's not six point seven exactly.”

P1

Participants shared their views on using a trusted information portal or digital library that could help them find useful online resources, personalized to their individual needs. A conversation among participants within focus group 2 highlights views on the potential benefits of an information portal or technology to build community.

I kind of love the idea of even like, I'm using this term really loosely, but like, an online library that has all of these resources that either our team has or other people have brought to them. … ‘cause you can Google stuff, but it's nice to feel like you're getting something from a source that's a little more legitimate.

P7

The staff and the nurses at the hospital, they seemed to have a lot of knowledge,…they are suggesting events, and readings and … articles, …that could benefit from being shared on such a portal.

P6

Throughout this discussion, responses were uniformly favorable toward the possibility of having an internet-based portal to find trusted information, with resources that were vetted by health care professionals and T1D peers going through similar challenges. Furthermore, the potential of using an app to build communit*y* posits benefits for participants in this discussion group, as it can act as an outlet to share first-hand T1D information and connect people with T1D within their local community.

### Establishing Trusting Relationships With Diabetes Care Providers Through Holistic Care

As participants gained experience and confidence with self-management, they said they relied less on their health care teams. However, most participants reported that they needed to check in periodically with their health care teams to keep them on track and help navigate new challenges. For instance, a participant compared their regular diabetes care visits to a “vaccine; [P17]” something they felt could prevent them from experiencing long-term complications. Another participant characterized their regular follow-up visits as a “wake-up call” [P19], which could keep them from becoming too complacent*.*

Participants also described the importance of building trusting relationships with their health care teams. As one participant described, their relationship with their nurses became like a “*borderline friendship* (P5).” Another participant highlighted the importance of psychosocial support given how self-management is “*entwined (P7)*” with everything else in their life,

The nature of being diabetic, it just gets wrapped up in everything in your life. So, I often think… my nurses … have to kind of be a therapist as well. (laugh) Because, when I go in and I say, like, you know, this week, or, 'These months have been bad, because… I lost my job.' or whatever the reasoning is. Like, it always ends up being about everything else that's going on in your life, because diabetes is so entwined in everything you do. And they are so supportive about that. And they just listen (laugh) and they let you… get your emotions out if you need to. And I find that incredibly helpful.

P7

Although participants emphasized that frequent interactions with providers helped them stay on course with their self-care, they also noted that these interactions represented only a fraction of the time they spent self-managing and navigating through the complex social contexts of everyday life. As one participant commented,

When I see my endocrinologist, it's very quick…I see her for ten minutes.

P24

### Perceived Benefits of Peer Support

Beyond the assistance offered by clinicians, participants spoke about the benefits of being involved with the T1D community and engaging with peer support. For instance, one participant explained that using social media allowed them to connect with other people living with T1D facing similar challenges:

And it's just more… quick, to get responses from social media, as opposed to getting appointments with specialists … Like, all these small issues that we deal with every day, not in textbooks that they [health care professionals] can't necessarily relate to. So it's good… to get different perspectives, from different people.

P8

Connecting with others living with T1D can be helpful for patients. Indeed, participants stated that they enjoyed participating in the focus groups and talking to others about their self-management experiences as it gave them an opportunity to share views and compare knowledge:

This is the first time I've been invited to something like this. So thank you, … But you know, it's a pleasant surprise that there's, obviously, other [people] who are dealing with the same potential struggles. And you know… I don't know very many diabetics, so it's nice to know that there are other [people], of similar age brackets, out there, that you know, are dealing with the same things that we're dealing with.

P4

Unlike a typical Facebook group, participants noted that our focus groups provided an opportunity for participants to interact (either virtually or in person) and learn from one another. This positive feedback regarding the focus groups reflects the potential benefits of incorporating more peer support programs into T1D care.

### Pre– and Post–COVID-19 Perspectives on Virtual Care

A central focus of discussions was on participant perspectives and experiences related to virtual care (ie, clinical care provided through phone or videoconference). Notably, the first 4 focus groups occurred before the onset of the COVID-19 pandemic and the enforcement of physical distancing measures in Canada (March 2020), which reflects a different context compared with the 2 later groups, when there was a far greater uptake of virtual visits for diabetes patients across Ontario. Throughout this section, we will consider virtual care across participant discussions, highlighting what may have changed due to COVID-19 or stayed the same.

Participants explained that the pandemic marked a time in which virtual visits became the new “normal,” mandated by public health measures. During the prepandemic focus groups, participants generally agreed that a shift toward more virtual visits made sense for diabetes care, and it was already “*going this way in the future* (P24)”

I did just have, actually, a virtual appointment. It was done over the phone…and it was perfect. It saved me a very long commute. (laugh) And, we accomplished all the same things, so it was pretty great.

P5

Aside from physical examinations, many participants said that most diabetes care could be done virtually, and they highlighted specific advantages of virtual care, such as saving travel time and reduced time off work. However, participants questioned what might be “lost” in virtual encounters:

What about the human interchange factor? We're not robots…There’s always something lost in translation… I'm not sure what would be lost yet … so it's good to have the option of both [virtual and in-person visits.

P17

Participants valued having in-person touch points with providers; physically being in the clinic made some participants feel as though they were more engaged with their care:

I find that when I go, I'm a hundred percent there, in mind and spirit. And you know, I feel a little bit more engaged.

P4

However, participants noted that having trusting relationships with healthcare providers that they have already met face-to-face in the clinic enhanced their engagement and satisfaction during virtual appointments:

I wouldn't mind the virtual appointments at all. Because I know my team. I've met them face to face…But, if I didn't know those people as well as I do right now, I would not be as happy doing it in a virtual environment.

P20

Overall, participants appeared to value the ability to contact providers when urgent issues arose between clinic visits. They clarified that they did not need providers to be at their “*beck and call*” (*P4*). Rather, it was important to know that they could get a timely response from a trusted health care provider when more unanticipated situations occurred related to their self-management.

## Discussion

### Principal Findings

We conducted focus groups with adults living with T1D in Ontario, Canada, to better understand their self-management experiences and how they viewed virtual care (comparing perspectives both before and after the pandemic). Participants represented a diverse group of adults with T1D from various life stages, occupations, and duration of diabetes. Our findings illuminate some common concerns, experiences, and needs of adults living with T1D at various life stages. These are important to consider, in this increasingly virtual era of diabetes care.

Looking beyond HbA_1c_ toward more nuanced indicators of glycemic management, participants noted that using “time in range” to identify glycemic patterns and focusing on physical symptoms associated with high or low blood glucose levels might be more valuable and practical for informing their self-management. Moreover, participants spoke about the critical role of incorporating technology within their lives to ease the burden of daily decision-making. Although participants generally spoke positively about advances in diabetes technologies (including insulin pumps, artificial pancreas, and looping systems [38]), some also expressed concern regarding information overload from the abundance of CGM data. Previous research suggests that information overload can decrease maintained use of CGM devices [10]. More research needs to be done to highlight ways in which diabetes technology can decrease the workload associated with T1D self-management rather than add to it.

Furthermore, participants spoke about the benefits of peer support, and leveraging the knowledge and skills of the T1D community, to learn how to adapt self-management education to evolving needs. This finding was similar to other studies in the T1D literature related to peer support; for instance, Elnaggar et al [18] found that the sharing of T1D experiences and first-hand knowledge through social media can serve as a catalyst for motivation and self-efficacy. Similarly, in our focus groups, there was also a general consensus that leveraging the support of the diabetes community (either online or in-person) was beneficial to participants by allowing them to connect with other people living with T1D who were going through similar life circumstances. Therefore, diabetes care teams may benefit patients by trying to find innovative ways to facilitate community-building amongst patients within their clinic.

In addition to engagement with peer support, mobile self-management interventions for people with T1D, have the potential to improve glycemic control when paired with input from clinicians (eg, through SMS text messaging or other communication modalities that promote frequent patient-provider interactions) [15-17]. Participants recounted that in order to establish trusting relationships with health care teams, diabetes care providers must try to individualize self-management education and to do this, mobile and online care may be the tool providers need to better connect with people with T1D and learn what their needs are outside of the clinic [15].

Participants in our study spoke at length about trying to find ways to adapt their diabetes self-management practices to their evolving lifestyle changes, which they described as an ongoing learning process. In doing so, they often used the language of “control”. Specifically, participants spoke about how the notion of trying to be “in control” of their blood sugars can feel burdensome, especially during challenging life periods. Given that the term “glycemic *control*” is widely used in the diabetes literature and in clinical practice, we have reflected on the use of this terminology throughout our qualitative analysis and in the writing of this manuscript after hearing from our patient partners. Using words such as “control” can leave individuals with feelings of guilt and being a “bad” patient when they are not reaching their target ranges or personal diabetes goals [5-8]. Moreover, the word “control” can invoke ideas of power struggles; for example, between parents and emerging adults [13], or people living with diabetes and their health professionals [14]. Our discussions with our patient partners about the language used in diabetes care and education reflect a greater movement in the literature toward being sensitive to issues of judgment (or even stigma) in patient encounters [5].

One of the key strengths of this project was using a patient engagement approach in all aspects of the study [24]. The conception, planning, design, analysis, and drafting of the manuscript were guided by people with lived experience of T1D. Collaboration with people with lived experience can improve the quality and relevance of research by focusing on the priorities set by patients [24]. Furthermore, the present qualitative study is informing the design of the broader T1ME trial [21] with the intent that engaging patients in the study design and selection of outcomes can lead to increased recruitment and retention in the trial, because study measures will be more meaningful and applicable to the lived experience of the target population [20].

Finally, there are some important limitations in our study that we would like to address. In terms of recruitment, we had to cease data collection after the sixth focus group as we were unable to continue recruiting participants in person at diabetes clinics due to the onset of the COVID-19 pandemic and local social distancing policies enacted in Ontario. Although some studies have shown that virtual recruitment may have benefits for qualitative research, such as the inclusion of a more diverse population [40], we found that it was much more difficult to recruit participants through advertisements and flyers compared with approaching them in person at clinics. We also acknowledge that there may have been an accessibility barrier for some individuals with T1D in Ontario (who may have trouble accessing technology) and these individuals may have different views compared with the perspectives shared within this study. Another limitation was that all but one of our participants lived in urban settings. Thus, our results may not fully reflect the experiences of those living in rural areas. Furthermore, the issue of “dominant voices” taking over a focus group discussion can be a concern for the interpretability of qualitative findings [41]. Finally, we appreciate diabetes care providers may have different views on the implementation of virtual care programs compared with patient participants. For instance, virtual care has been reported to increase the workload for nurse educators in some telehealth programs [42]. Therefore, it is important to consider what additional support may needed for health care providers while implementing virtual health care interventions.

### Conclusion

Type 1 diabetes care has shifted toward a more virtual model of care that is in line with the unique needs and preferences of a diverse patient population with T1D who require personalized education and timely support to help manage a relentless and complex chronic condition. In our study, we have highlighted the perspectives of people with T1D in Ontario who are trying to adapt to this more virtual landscape of diabetes care in Canada. Our findings help explore the ways in which people with T1D want technology to meet them “where they are at” in their unique T1D journey and provide an opportunity to think about more patient-centric outcome measures that go beyond HbA_1c_, such as symptom control (alleviating fluctuations in highs or lows) and the patient perceived benefits associated with time-in-range. Although participants generally spoke positively about technological advancements in CGM and insulin delivery systems (CSII), there were contrasting perspectives related to the issue of information overload, which sheds light on the need for additional support to navigate the increasingly data-driven nature of T1D self-management. Our findings indicate that finding ways to use technology to leverage the provision of personalized support of peers, as well as providers, can help build a sense of community, and bridge the gap between the clinical care needs of individuals with T1D and the complex social context that surrounds their daily glycemic management.

## Appendix

Multimedia Appendix 1 Focus Group Guide.

## Abbreviations

CGM: continuous glucose monitoring
HbA_1c_: hemoglobin A_1c_
PAC: Patient Advisory Committee
PREM: patient-reported experience measure
PROM: patient-reported outcome measure
T1D: type 1 diabetes
T1ME: Type 1 diabetes virtual self-Management Education and support
