# Supplementary material for: Beyond Hemoglobin A1c—Outcomes That Matter to Individuals With Type 1 Diabetes in Adopting Digital Health Interventions for Self-Management Support: Qualitative Study
Source: JMIR Diabetes. 2024 Nov 7;9:e60190. doi: 10.2196/60190 (PMC11582490; doi:10.2196/60190)
Supplement: Multimedia Appendix 1 [file diabetes_v9i1e60190_app1.docx]

**Appendix 1 - Focus Group Guide**

**Introduction/Icebreaker**

I would like you to introduce yourselves. Can each of you tell the group how long you have been living with Type 1 diabetes and what made you decide to join this discussion group today?

**Personal experiences managing Type 1 diabetes**

1. What do you think about related to your Type 1 diabetes self-management during the course of a typical day?
   - Probes: changing insulin pump sites, hypoglycemia or hyperglycemia, etc.
     - Does anything bother you about self-managing Type 1 diabetes?
     - Does your diabetes management ever interfere or intrude with other areas of your life (e.g. work, family, education)?
2. When you are self-managing your diabetes, what are you hoping to achieve? What goal(s) do you have?

- Why are these important for you?
- Do you have difficulty attaining these goals? Can you tell me about that?
- What is or would be helpful in assisting you to achieve these goals?

1. What is your relationship like with your health care providers?
   - How do they help with self-management?
   - Are there any specific types of health care providers you feel closer or less close with? Why?
   - How do you communicate with your health care team between visits? (e.g. email, text, phone, etc.).
   - What would be an ideal way of communicating with your health care team?
   - What would the ideal frequency of communication be with your health care team? Probes: daily, weekly, as needed (for example, when crises arise or you encounter a new challenge in your life).
   - What are your thoughts on virtual care visits? Do you think these would be helpful for your self-management needs?
   - Are there any health care supports that you are not currently receiving, but think would be helpful in managing your diabetes?
2. Outside of the clinic, are there people or other information resources you use to support your self-management?
   - Where do you go to find trusted information related to your management? Do you ever find you need more support or guidance for this?
   - What role, if any, do friends and family play?
   - Do you ever feel like you need more support outside of the clinic context? Can you tell me about that? Can you provide an example?

**We are about to transition into a discussion about your use of apps generally, and health management/diabetes management apps specifically. Before doing so, is there anything else that you would like to discuss about your experiences with diabetes management?**

1. I wanted to get a sense of the group’s experiences with apps. Perhaps we could begin by discussing if you use apps and if so, which apps you tend to use most?

Probes:

- Why do you use that app? What features of the app do you find helpful? Are any features unhelpful?
- Does anyone use health management apps? Which ones? Can you tell me about that? Do you use any diabetes related smartphone applications to help with self-management? Tell me about your experiences with these.
- If you do not use health management or diabetes management apps, can you tell me why?

**We would now like you to apply these discussions to a hypothetical situation: Technology Company wants to know if they improved a patient’s quality of life**

1. We would like to present a hypothetical situation to you. In this situation, a technology company has created a new smartphone application that intends to help improve the lives of people with Type 1 diabetes. However, none of the app developers has experience self-managing Type 1 diabetes and they need advice from those that do. One of the primary questions they have for you is, *if you were to use this application for a prolonged period of time, what would make you feel as though self-management became easier for you?*

Potential probes:

- - What functions would you like to see in the app? Can you tell me about why those would be important for you? What impact would this have on your diabetes self-management?
  - What changes in your life would you like to see and why?
  - What would make you feel as though this self-management support tool was a success? What would make you feel as though it failed to make improvements?
  - How could an app that allows videoconferencing with your healthcare providers address patients’ self-management needs?

7. We are coming to the end of our discussion today. However, before we conclude, is there anything else that you wish to add? Anything we haven’t discussed that you think is important for us to know?
